# Occupational physical activity: the good, the bad, and the proinflammatory

**DOI:** 10.3389/fmed.2023.1253951

**Published:** 2023-10-06

**Authors:** Galateja Jordakieva, Timothy Hasenoehrl, Margarete Steiner, Erika Jensen-Jarolim, Richard Crevenna

**Affiliations:** ^1^Department of Physical Medicine, Rehabilitation, and Occupational Medicine, Medical University of Vienna, Vienna, Austria; ^2^Center for Pathophysiology, Infectiology and Immunology, Institute of Pathophysiology and Allergy Research, Medical University of Vienna, Vienna, Austria; ^3^The Interuniversity Messerli Research Institute, Medical University of Vienna, Vienna, Austria

**Keywords:** inflammation, proinflammatory markers, high activity work, sedentary work, occupational health

## Abstract

**Background:**

Physical activity (PA) is beneficial for preventing several conditions associated with underlying chronic inflammation, e. g., cardiovascular disease (CVD) and cancer. While an active lifestyle appears to have anti-inflammatory effects, high levels of occupational PA (OPA) were associated with inflammation and elevated mortality risks. We aimed to summarize the current knowledge (1) on the association between inflammation and OPA and (2) its implications for health and mortality.

**Methods and results:**

This mini-review summarized relevant literature published before January 2023 using established scientific databases and sources. For the primary outcome, observational studies (S) reporting immunological effects (O) in subjects (P), with high (I) vs. low OPA (C), were included. For secondary outcomes, i.e., morbidity and mortality associated with inflammatory processes, (systematic) reviews were included. While “active” occupations and “moderate” OPA appear to have beneficial effects, low (particularly sedentary) and “high-intensity” OPA (particularly including heavy lifting tasks) were associated with inflammation and (CVD and cancer-related) mortality; higher leisure-time PA has been almost consistently associated with lower proinflammatory markers and all-cause mortality risks. Workplace interventions appear to counter some of the observed health effects of unfavorable work strain.

**Conclusion:**

The few studies addressing OPA “intensity” and inflammatory markers are largely heterogeneous regarding OPA classification and confounder control. Sedentary and “heavy” OPA appear to promote proinflammatory effects. In addition to targeted management of work-related physical strain and hazardous environmental co-factors, occupational health providers should focus on employer-initiated exercise interventions and the promotion of leisure-time PA.

## 1. Introduction

The benefits of physical activity (PA) for health and longevity have been well established. PA has been associated with the prevention and management of excessive body weight, chronic disabilities, and health conditions [e.g., cardiovascular disease (CVD), metabolic syndrome, cancer], strengthening of the musculoskeletal system, and improvement of cognitive functioning and mental health (1). Chronic (low-grade) inflammation is involved in the pathogenesis of several conditions mentioned, such as atherosclerosis, metabolic dysfunction, the development and promotion of cancer, and autoimmune and neurodegenerative diseases (2). Although the exact mechanisms are largely undefined, the growing field of exercise immunology describes how PA is involved in immune modulation and how an active lifestyle mediates anti-inflammatory and antioxidant states, refining dysbiosis of the immunologically highly active gut microbiome and countering the development of chronic health conditions as well as immunosenescence (3). A history of physical inactivity has been associated with an increased “immune risk profile” based on biomarkers predicting morbidity and mortality in the elderly (4). Accelerated aging, cognitive decline, and impaired vascular and immune functions have been associated with an inactive and particularly sedentary lifestyle (5). Immune-specific findings of physical inactivity and sedentary lifestyle include *in vitro* observations regarding the shortening of leukocyte telomere length, proinflammatory immune mediator production, and impairing innate and adaptive immune cell activity, as well as *in vivo* responses to inflammatory processes (4). While widely unspecific, circulating inflammatory markers, such as high-sensitivity C-reactive protein levels [(hs)CRP], have been associated with pre-diabetic status (6) and have emerged as more reliable indicators of atherosclerosis than classical lipid markers, e.g., low-density lipoprotein (LDL) cholesterol (7) in CVD. Biomarkers of chronic and systemic inflammatory responses have also been identified as independent prognostic factors, particularly in CVD and cancer-related mortality risk (8). In this context, PA was found to exert beneficial effects on mortality risk associated with a high systemic immune-inflammation index.

A meta-analysis including data from over 122,000 participants (60 years+ of age) reported a curvilinear relationship between overall weekly moderate to vigorous PA [based on metabolic equivalents of task (METs)] and all-cause mortality, with a steep initial increase in benefits and a hereafter linear reduction of mortality from medium to high doses of moderate to vigorous PA. The authors described the strong inverse relationship as related to reduced CVD and, to a lesser extent, reduced cancer-related mortality (9). In 2018, however, a meta-analysis including data from 193,696 participants reported that, compared to low levels of occupational PA, high levels of occupational PA significantly increased mortality risk in men (10). These findings intensified the debate over the existence of a “physical activity paradox,” which indicates that higher levels of leisure-time PA but not occupational PA are beneficial to health, an effect possibly mediated through proinflammatory processes associated with high levels of occupational PA, specifically (11). This mini-review aimed to summarize what is currently known about inflammation in the context of occupational physical activity (OPA) and the potentially associated implications for health, morbidity, and mortality.

### 1.1. Search strategy and manuscript selection

This mini-review aims to identify available evidence on the immune effects of occupational physical activity (12) via PubMed (MEDLINE), Embase, and the Cochrane Library using the keywords (“occupation^*^” OR “work-related”) AND (“physical activity”) AND (“inflammation” OR “immune”). All article types (e.g., original articles, reviews, meta-analyses, editorials, and letters) from inception to 15 January 2023 were screened by abstract regardless of language. Studies were included and evaluated according to the PICOS framework: any observational (cohort, case-control, cross-sectional) studies (S) reporting immune effects (O) in (currently or previously) working subjects (P), with high occupational physical activity (I) vs. controls with low physical activity (C), were included for evidence analysis. The PICOS tool, endorsed by the Cochrane Collaboration, focuses on (P)opulation, (I)ntervention, (C)omparison, (O)utcomes, and (S)tudy design in quantitative research; it is conducive to the identification of relevant components of clinical evidence in scientific reviews (13). Relevant studies were further identified by screening (systematic) reviews and meta-analyses. Nine studies reporting changes in inflammatory markers in relation to OPA activity levels were identified (11, 14–21) and included in the narrative analysis.

Secondary outcomes of interest were morbidity and mortality, particularly outcomes linked to chronic diseases associated with underlying sustained inflammatory processes; here, systematic reviews were preferred in view of limitations on the number of references in a mini-review.

## 2. Occupational physical activity and inflammation

### 2.1. Low to moderate OPA: the good?

Exercise, but also non-exercise PA, such as occupational or household work, has been reported to result in lower levels of circulating CRP, interleukin 6 (IL-6), and tumor necrosis factor-alpha (TNF-α) in the elderly (15). Moderate weekly PA, regardless of leisure or occupational context, was found to correlate with the modulation of circulating inflammatory markers (16). Total PA was associated with lower proinflammatory TNF-α expression and higher anti-inflammatory IL-10 expression in breast tissues of physically active women, as compared to women in the lowest PA categories; higher OPA was inversely associated with a pro- to anti-inflammatory mediator ratio among premenopausal women in an adjusted model, with a particularly low expression of both IL-6 and TNF-α in women with high OPA (17). Higher levels of overall PA, including occupational activity, were associated with reduced circulating CRP and malnutrition-inflammation scores in hemodialysis patients (18).

Moderate and higher OPA resulted in decreased ischemic heart disease risk and were associated with lower CVD mortality rates in women (22) and men (23, 24). An “active” job showed modest associations with a reduced total incidence of stroke, specifically with a lower ischemic stroke risk in both sexes combined; in this context, active commuting by bicycle or on foot (≥30 min per day) also showed a moderate protective effect. Combining moderate and “active” OPA vs. low OPA resulted in reduced adjusted stroke risk, even after controlling for leisure time and commuting PA (25). Previous smaller studies did not find an association between stroke risk and OPA (26, 27). Moderate to vigorous OPA was significantly associated with a lower diabetes type II risk after adjustment for common risk factors and general (commuting and leisure-time) PA (28). In chronic inflammation-based conditions, such as inflammatory bowel disease (IBD), occupations characterized by higher OPA had protective effects (29) regarding the risk of contracting IBD compared to those with less active OPA (30). A meta-analysis further showed that a significantly reduced colon cancer risk was associated with higher leisure-time PA and higher OPA in men, whereas in women, only leisure-time PA was associated with a reduced colon cancer risk (31). While in another meta-analysis, higher levels of OPA were associated with decreased bladder cancer risk, this finding only reached statistical significance after combining risk estimates with leisure-time PA (32). A protective effect of an “active” job was also hypothesized for breast cancer (33, 34); circulating leukocyte telomere length, a marker for cell aging of innate immune cells involved in anti-cancer responses, was positively associated with overall PA and OPA, respectively (35).

### 2.2. Low occupational physical activity: the bad?

A sedentary lifestyle in combination with elevated hs-CRP appears to have a particularly high all-cause, CVD, and cancer-related mortality risk (14). An inactive lifestyle, i.e., a sedentary job and no recreational activity, was associated with the highest CRP levels and a higher risk of future coronary artery disease in a large case-control study, compared to higher OPA levels partially even without recreational activity (21).

In a prospective study including over 1,500 participants, sedentary OPA was described as a risk factor for all-cause mortality with an adjusted hazard ratio of 1.16 and independent of overall PA, with the lowest cumulative survival rates in occupational groups with the highest sedentary time compared to more active OPA groups (36). Association with mortality remained after adjustment for total PA and common confounders such as age, sex, body mass index, total cholesterol levels, and (systolic) blood pressure. These findings were observed in both genders but did not reach statistical significance for women (36). Low levels of OPA and leisure-time PA were independently associated with higher mortality risk in a Belgian Physical Fitness Study in 1,456 men (37); a sedentary lifestyle was proposed to be more harmful to health in workers with lower overall physical fitness (37). One study validated reported occupational sitting time applying accelerometry and found that sedentary behavior at work was not associated with CVD events and/or mortality, a risk when adjusted for confounders (38). In this study (36), sedentary OPA was significantly associated with CVD-related but not cancer-related mortality, whereas a meta-analysis described a positive association between occupational sedentary time and colon cancer risk, specifically (39). Low OPA, particularly sedentary OPA (40), was also associated with a higher risk of developing IBD in contrast to more active jobs. Not surprisingly, a proinflammatory diet was associated with sedentary OPA in a cohort of Croatian workers (41).

### 2.3. “High-intensity” occupational physical activity: the proinflammatory?

Increased levels of hs-CRP were recently associated with higher OPA and lower levels of leisure-time PA, suggesting an association between systemic inflammation and the “physical activity paradox” (11). The highest levels of OPA were proposed to be directly associated with higher circulating hsCRP and thus an increase in systemic inflammation (19); workers engaging in “higher-intensity” OPA had significantly higher hsCRP levels compared to those with lower OPA and high levels of leisure-time activity, with sex-stratified models again showing more significant outcomes in men than in women. Another study found no statistically significant relationship between “work-related strain” and inflammatory markers (20) but between OPA and CVD risk.

The highest levels of OPA were reported to increase the risk of all-cause mortality, particularly in men (42). Associated increases in blood pressure, prolonged elevation of heart rate (43), and sustained levels of inflammation (44) were proposed as mediating risk factors for CVD in “high-intensity OPA” (45, 46). A significant increase in mortality was observed in workers with high OPA, based on job type and kilocalories per working hour, and low leisure-time PA after adjustment for potential confounders, particularly in those with lower overall physical fitness (37). Strenuous OPA, but particularly repetitive and heavy lifting at work, was associated with an increased myocardial infarction risk (47); “high-intensity” OPA was associated with CVD risk in women (48). A positive association between OPA “intensity” and stroke/transient ischemic attack (TIA) was described after controlling for confounding factors. An increased risk was found for “partially standing” and “high-intensity work,” with a dose-response relationship between stroke and exposure at the longest-held job but also between TIA and current job; leisure-time PA was again described as protective against stroke and TIA (49).

Particularly, heavy lifting tasks are a special aspect of more intensive OPA and are commonly ascribed to the highest OPA categories. Musculoskeletal pain, overuse injuries, and knee osteoarthritis have also been associated with high levels of OPA, particularly heavy lifting and repetitive movements (50). While circulating inflammatory markers are released following tissue damage (51, 52), potentially predicting overuse injuries, some inflammation is considered necessary in the initiation of favorable tissue repair and physiological adaptations to increased working demands (53). Interestingly, proinflammatory mediators, such as IL-6 and IL-8, are increased with higher body fat percentage and in response to lifting work, with low-frequency high-resistance tasks associated with higher systemic inflammatory responses and greater cumulative spinal moments compared to high-frequency low-resistance tasks (51).

## 3. Discussion

While overall PA and an “active” occupation appear to be associated with improved inflammatory markers and related morbidity and mortality risks, the outcomes for very low and sedentary OPA are inconclusive or unfavorable, especially when combined with low leisure-time PA. Emerging evidence points toward the proinflammatory effects of “high-intensity” OPA. Overall, due to the heterogeneity in the design of the few available studies (11, 14–21), still very little is known about the association between OPA “intensity” and inflammatory markers, which are easily impacted by pre-existing comorbidities and immune-modulating medication, but also by (blood) sampling timepoints in relation to diurnal rhythm and hours since PA (54, 55).

Proposed reasons for the frequently reported lack of health benefits of OPA in contrast to leisure-time PA are the suboptimal design of OPA for improving cardiorespiratory fitness, particularly in terms of (potentially harmful) working posture, intensity, or duration of working tasks; furthermore, uncontrolled elevation of 24 h heart rate and blood pressure (particularly by heavy lifting work), lack of sufficient recovery time between intensive tasks, and limited control over work-associated physical and psychosocial stressors (45) are potential proinflammatory hazards. Interestingly, <35 working hours per week were associated with lower all-cause mortality, independent of gender and OPA intensity (56). In addition to long working hours and shift work, which are found in all OPA categories, other working environment factors with proinflammatory potential, such as heat strain, are more common in “high-intensity” OPA jobs (57–59). Lack of sufficient recovery time (60) in combination with work-related and work-independent stressors may indeed support sustained inflammation and promote the development of chronic diseases, such as CVD and cancer. Age, gender, and potentially race are factors associated with differences in the expression of inflammatory markers (61) and probably also with the extent of health benefits derived from PA in general (10).

### 3.1. Current controversies and research gaps

Valid criticism (62) has also been raised against the occupational “physical activity paradox,” including the often imprecise and usually self-reported determination of “active” and/or “high activity” occupational tasks using questionnaires only. One review summarizing device-measured PA at work reported that while office workers had primarily sedentary OPA, they still were most active during their day compared to “more active” professions such as healthcare workers and laborers (63). Furthermore, the differentiation between OPA “intensity” varies between most studies, and categories of OPA are sometimes merged to obtain larger subgroups. Here, a consistent classification (64), ideally based on objective work intensity data including METs (65) and accounting for work-specific conditions, e.g., heavy lifting tasks and working environment, should be agreed upon for the design of further studies. At this point, we also need to add that OPA is always “physical” work. This should be taken into consideration because, from the perspective of physical work, in OPA, there is currently no differentiation between workload intensity, volume, or density. Although the use of METs makes sense for the objective representation of workload, we still do not understand “high intensity” OPA well enough. Is it truly work “intensity,” and therefore the very high workload concerning the worker's maximum strength, which leads to detrimental health outcomes? Or is it work “volume” and therefore the extensive sum of cumulative workloads? Or is it maybe work “density” and therefore the lack of sufficient rest periods between phases of increased physical work?

The choice of relevant co-variates has also been argued as a potential bias, e.g., the gradation of confounders such as smoking (62). Additionally, commonly controlled confounders are imprecise, such as BMI, which does not adequately reflect muscle vs. fat-free mass, which again makes a significant difference in terms of inflammatory potential and physical resilience. Lastly, synergistic and potentiating effects of confounders, such as multiple unhealthy lifestyle behaviors, including proinflammatory diet consumption at the workplace, commonly associated with, e.g., social class, cannot be fully controlled for and might affect certain “heavy-work” OPA groups more than other OPA categories (66). While workplace safety has been considerably improved over the last decades, studies addressing all-cause mortality among heavy workers should also consider specific hazards of the working environment, e.g., the impact of occupational diseases and fatal accidents (67).

### 3.2. Potential future developments in the field

**Figure 1:**
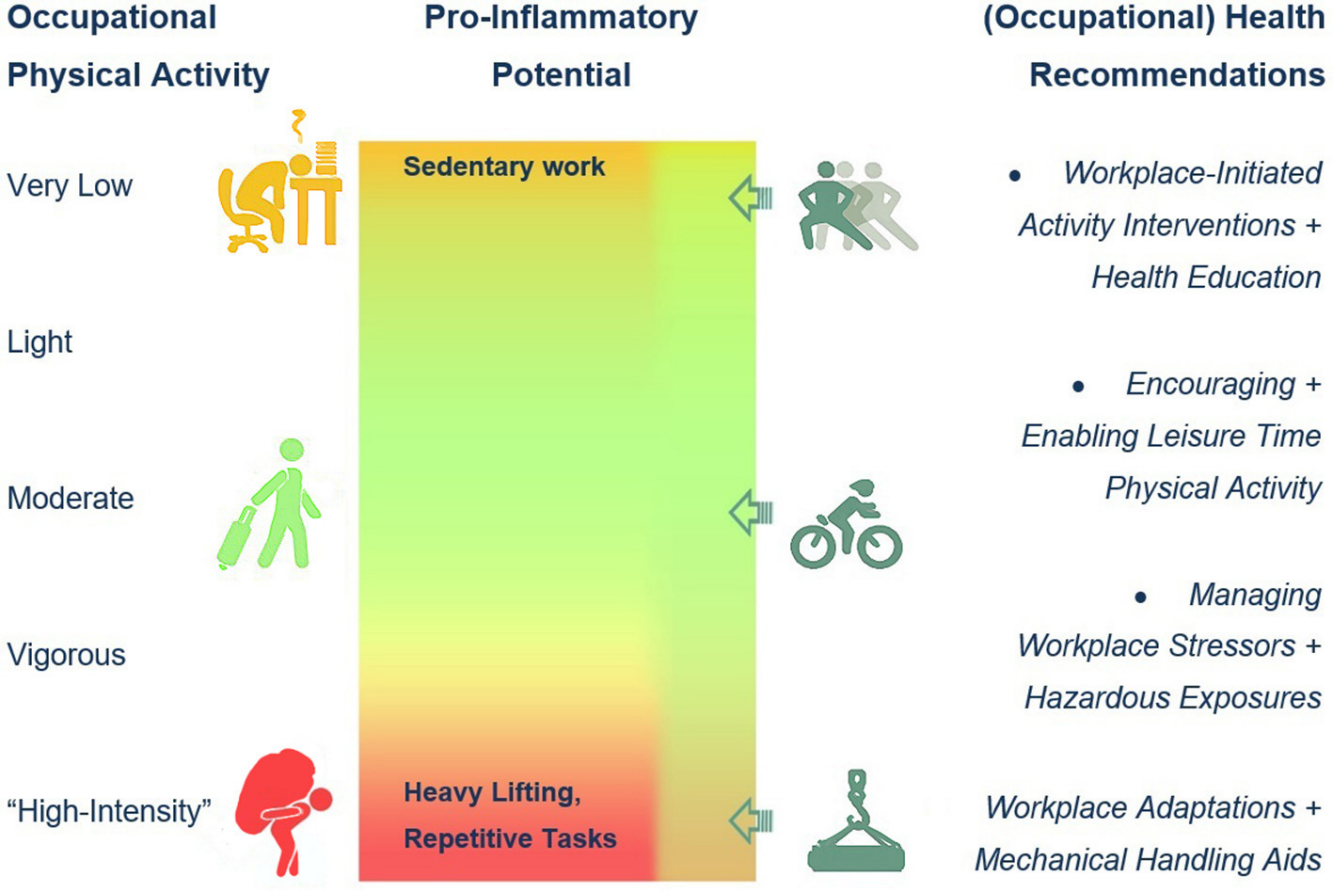
Occupational physical activity (OPA) levels, suspected proinflammatory effects, and occupational health recommendations.

Several strategies for improving health in potentially hazardous high OPA environments have been proposed (45), such as regulating task intensity and recovery time throughout the working day, using mechanical support equipment to avoid heavy manual lifting, and delivering activity interventions through the workplace (Figure 1). Leisure-time PA has been repeatedly described as protective against potentially inflammation-mediated morbidity and mortality, even in workers with unfavorable (sedentary or “high-intensity”) OPA. Simple occupational health promotion, such as activity and walking interventions and even unsupervised employer-initiated PA (68), workplace stressor handling, including a healthy diet and reduction of hazardous stressors, as well as the introduction of mechanical handling aids in the case of heavy OPA (Figure 1), may sustainably improve inflammatory profiles (69) and potentially reduce CVD risk (70).

## 4. Conclusion

While proinflammatory effects have been originally attributed to very low and sedentary OPA, “high-intensity” OPA is emerging as a risk factor for chronic inflammation and potentially associated mediated morbidity and mortality risks. There is still very little known about the link between OPA “intensity” and inflammatory markers, mainly due to the heterogeneity of OPA classification and confounder control in the available studies. Although the existence of the “physical activity paradox” is still debated, a consensus regarding the necessity for (a workplace-mediated) encouragement of leisure-time PA is evident throughout the scientific literature and arguably for an ever-continuing optimization of workplace conditions in accordance with occupational health care and promotion (Figure 1).

## Author contributions

GJ: Writing—original draft, Writing—review and editing. TH: Writing—review and editing. MS: Writing—review and editing. EJ-J: Writing—review and editing. RC: Writing—review and editing.
